# Harmonized psychiatric diagnosis data from the Adolescent Brain Cognitive Development Study

**DOI:** 10.21203/rs.3.rs-10083024/v1

**Published:** 2026-06-19

**Authors:** Eugenia Giampetruzzi, Daniel S. Pine, André Zugman, Ian H. Gotlib

**Affiliations:** Stanford University; National Institute of Mental Health; National Institute of Mental Health; Stanford University

**Keywords:** ABCD, KSADS, diagnosis

## Abstract

In the Adolescent Brain Cognitive Development (ABCD) Study, the computerized Kiddie Schedule for Affective Disorders and Schizophrenia (KSADS-COMP) was administered to 11,858 participants at up to eight waves. The structure of these data creates the need for many scoring decisions. Present and lifetime diagnoses, and parent and youth reports, are each stored separately; two instrument versions are pooled into shared columns; the same administrative codes represent several distinct forms of missingness; branch-skip logic produces missing data by design, and modules were added and dropped across the biennial schedule. Researchers resolve these features differently, so definitions of diagnoses vary significantly across studies. The consequences are substantial: holding the data fixed at release 7.0, the prevalence of any single disorder in ABCD ranges from 1.9% to 56.8%, depending on which definition is used. In this paper, we present a pipeline that makes each of these decisions explicit, along with a harmonized, analysis-ready diagnostic dataset for ABCD release 7.0. We also provide recommendations for multiverse analysis and a clinically informed recommended configuration.

## Background & Summary

**Figure 1:**
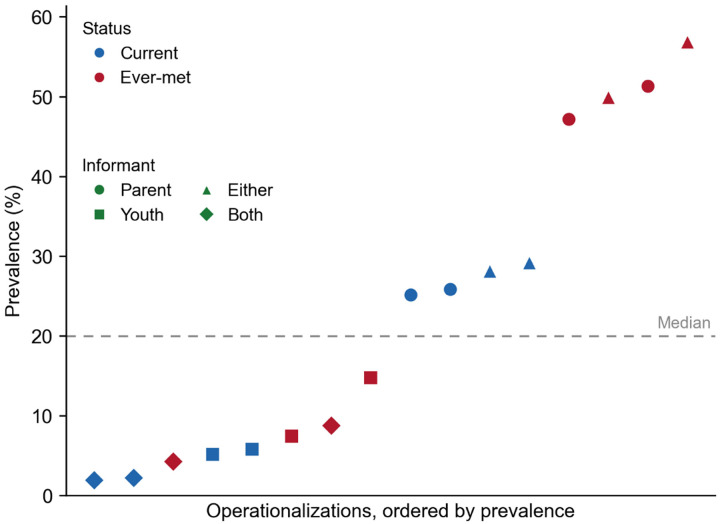
Diagnosis prevalence range across operationalizations *Note*. Each dot is one operationalization of “any disorder,” ordered along the x-axis from lowest to highest prevalence. The y-axis is prevalence (%), computed over administered cells only. Color denotes episode status (current vs. ever met), and shape denotes the informant rule (parent, youth, either, or both). The dashed line marks the 20% median across operationalizations.

The Adolescent Brain Cognitive Development (ABCD) Study is the largest longitudinal study of brain development and health in youth in the United States, following nearly 12,000 children from ages 9–10 years into adolescence^1^. Data concerning psychiatric diagnoses were obtained by administering the computerized Kiddie Schedule for Affective Disorders and Schizophrenia (KSADS-COMP^2^) to participants. These have been analyzed in a large and growing literature using ABCD data to examine associations of mental health with the brain, cognition, genetics, and the environment^3^. Analyzing the KSADS-COMP diagnostic data, however, requires a chain of decisions: how to understand administrative codes; whether to count past episodes alongside current episodes; how to combine parent and youth report. Each choice, on its own, can be reasonable; however, these choices are made inconsistently by different researchers across analyses. The result of this analytic “flexibility” is that specific diagnostic criteria, and the prevalence associated with each diagnosis, change across studies and across data releases; moreover, the consequences of these decisions are rarely reported. In this context, when we hold the data fixed at release 7.0, results show that the prevalence of any single disorder ranges from 1.9% to 56.8%, a nearly 30-fold range that is produced entirely by processing choices applied to the same data obtained from the same children (Fig. 1).

## Diagnosis prevalence range across operationalizations

### Note

Each dot is one operationalization of “any disorder,” ordered along the x-axis from lowest to highest prevalence. The y-axis is prevalence (%), computed over administered cells only. Color denotes episode status (current vs. ever met), and shape denotes the informant rule (parent, youth, either, or both). The dashed line marks the 20% median across operationalizations.

**Figure 2:**
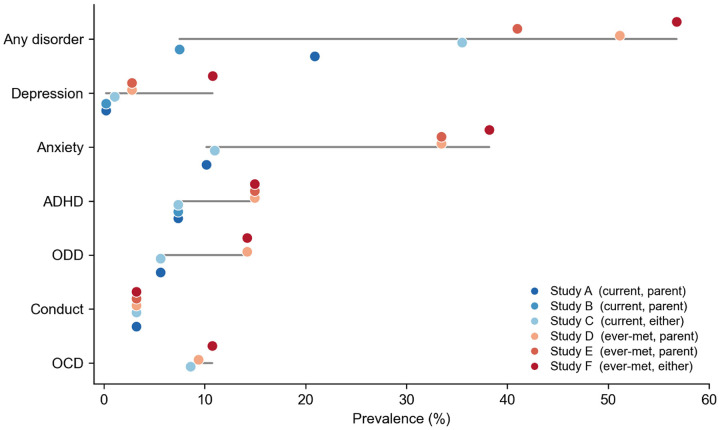
Diagnosis prevalence across six published operationalizations *Note*. Each row (y-axis) is a DSM diagnostic category; the x-axis is prevalence. Each dot is one of six published studies’ operationalizations (color denotes study, per legend; current vs. ever-met studies are grouped in blue and orange red, respectively). The horizontal line spans the range of estimates within a category, labeled with the fold-change between minimum and maximum. Study labels are anonymized (Studies A-F, 2021–2026).

This concern is not merely hypothetical. When we attempted to reproduce the operationalizations of six recently published ABCD studies^4–9^ on the same baseline data (ses-00A; N = 11,705 − 11,812, depending on the informant rule), prevalence estimates for the same diagnosis differed several-fold across studies (Fig. 2).

## Diagnosis prevalence across six published operationalizations

### Note

Each row (y-axis) is a DSM diagnostic category; the x-axis is prevalence. Each dot is one of six published studies’ operationalizations (color denotes study, per legend; current vs. ever-met studies are grouped in blue and orange red, respectively). The horizontal line spans the range of estimates within a category, labeled with the fold-change between minimum and maximum. Study labels are anonymized (Studies AF, 2021–2026).

**Figure 3:**
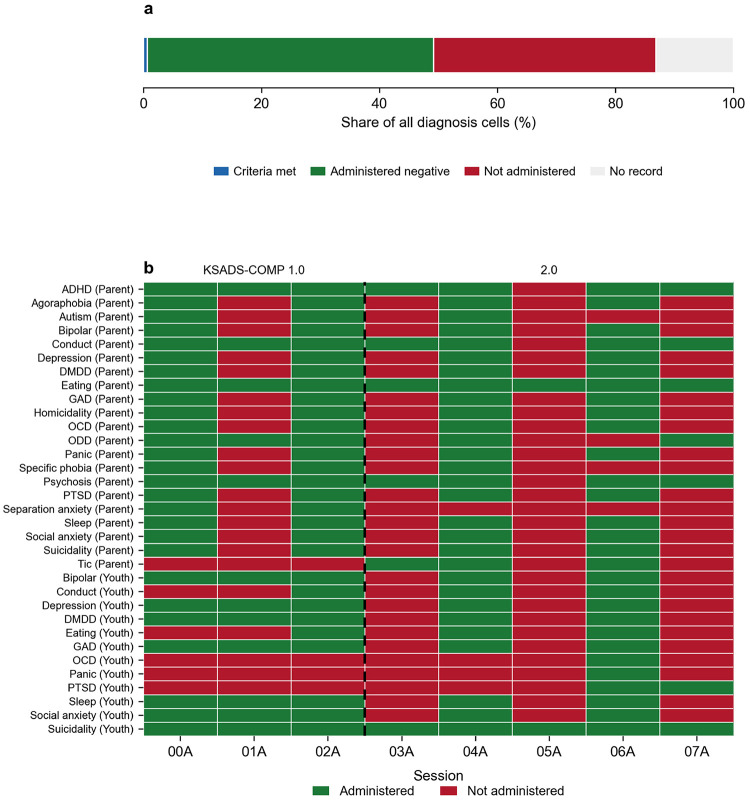
Administrative codes and module schedule *Note*. (A) Distribution of the four resolved cell states across all participant x session x diagnosis cells: criteria-met (0.7%), administered-negative (48.5%), not-administered / 555 (37.7%), and no-record / blank (13.2%). (B) Administration calendar showing, for each module (rows) and session (columns), whether the module was administered, not administered (present in the release but recorded entirely as 555), or absent (not in the release at that wave), separately for parent and youth report. The calendar marks the full biennial battery (baseline and the 2-, 4-, and 6-year follow-ups), the reduced interim annual batteries, and modules added or dropped mid-study.

There are several structural features of the data that make such divergence likely, if not inevitable, and each is a point about which careful analysts can disagree. The first, and perhaps most consequential feature, concerns missingness. The data use distinct codes for responses that were not administered (555), branch-skipped by design (888), and truly missing; states that are often collapsed into a single “screened-negative” code. The 555 code alone accounts for 37.7% of the roughly 18 million diagnosis cells in the wave 7.0 release, so reading each data point as “screened-negative” translates millions of never-assessed observations into “healthy,” deflating prevalence estimates and biasing longitudinal models that treats these youth as true negatives (Fig. 3). The second concerns the nature of form of the assessments. Present and lifetime diagnoses are stored as separate variables that researchers either combine, choose between, or treat interchangeably. Amplifying this concern, the KSADS-COMP changed from version 1.0 to 2.0 at the three-year follow-up (ses-03A), and the release pools both versions in shared columns. The assessed diagnoses are not constant across waves, as modules were added and dropped on different schedules (Fig. 3).

## Administrative codes and module schedule

### Note

(A) Distribution of the four resolved cell states across all participant x session x diagnosis cells: criteria-met (0.7%), administered-negative (48.5%), not-administered / 555 (37.7%), and no-record / blank (13.2%). (B) Administration calendar showing, for each module (rows) and session (columns), whether the module was administered, not administered (present in the release but recorded entirely as 555), or absent (not in the release at that wave), separately for parent and youth report. The calendar marks the full biennial battery (baseline and the 2-, 4-, and 6-year follow-ups), the reduced interim annual batteries, and modules added or dropped mid-study.

**Figure 4:**
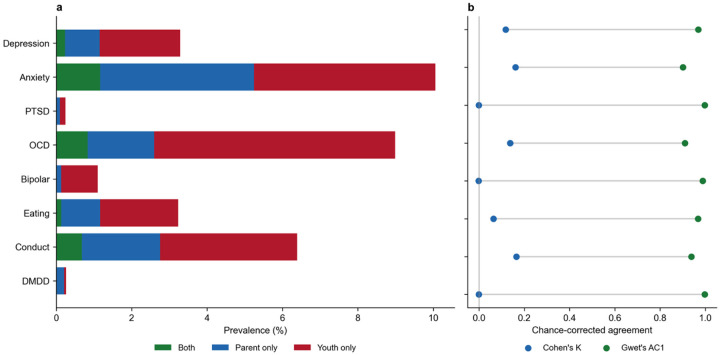
Informant discrepancies *Note*. Parent-youth diagnostic discrepancy at the Year 6 follow-up (ses-06A), restricted to the eight categories administered to both informants (N ≈ 9,098 assessed by both). (A) Each row is a DSM diagnostic category; the x-axis is prevalence. Each bar is split into cases endorsed by both informants, by the parent only, and by the youth only; total bar length is the ’either’ prevalence and the dark segment is the ’both’ prevalence. (B) Each row is a category; the x-axis is chance-corrected parent–youth agreement, shown as Cohen’s κ and Gwet’s AC1.

The third concern involves the ways that raw responses are translated into diagnoses. Parents and youth frequently disagree about the presence of a diagnosis (or even a symptom); this disagreement can arise from measurement error as well as meaningful differences between informants’ observations^10^. Consistent with this multi-informant literature, parent and youth report in ABCD disagree markedly. At baseline, Cohen’s κ is 0.02 for both depression and anxiety; for depression, only a single child was positive on both reports. Agreement remains low at later waves (κ = 0.06–0.17 across the eight categories administered to both informants at Year 6; Fig. 4). Unfortunately, there is no standard rule for reconciling parent and youth reports: a participant may be counted as a case based on the parent’s report, the youth’s report, on either or on both reports. Further, symptom-level information, along with current / past / remitted status, distinctions that have significant clinical meaning and implications, are often reduced to a single binary code.

## Informant discrepancies

### Note.

Parent-youth diagnostic discrepancy at the Year 6 follow-up (ses-06A), restricted to the eight categories administered to both informants (N ≈ 9,098 assessed by both). (A) Each row is a DSM diagnostic category; the x-axis is prevalence. Each bar is split into cases endorsed by both informants, by the parent only, and by the youth only; total bar length is the ’either’ prevalence and the dark segment is the ’both’ prevalence. (B) Each row is a category; the x-axis is chance-corrected parent–youth agreement, shown as Cohen’s κ and Gwet’s AC1.

To make decisions about the use of the ABCD KSADS-COMP data explicit and reproducible, we provide a processing pipeline together with a harmonized, analysis-ready diagnostic dataset for ABCD release 7.0 (230 diagnoses mapped to 16 DSM categories, 11,858 participants, all eight sessions), deposited in the NIMH Data Archive. For analysts who need a single definition, we provide a clinically informed recommended configuration. We also include a reporting checklist, paired with the specification-curve approach of Fig. 1, so that in future, investigators can state their diagnostic choices and show the sensitivity of their results to these decisions.

## Methods

### Overview.

We built the harmonized dataset from the tabulated KSADS-COMP data in ABCD release 7.0 (parent report = mh_p_ksads; youth report = mh_y_ksads). These contain 230 diagnosis variables across 21 parent and 12 youth modules, recorded over eight sessions (ses-00A, baseline, through ses-07A, the 7-year follow-up). The pipeline had six steps, each addressing one of the structural features described above: (1) resolving the administrative codes; (2) preserving episode status; (3) recording the instrument version; (4) documenting the administration schedule; (5) specifying the informant(s); and (6) assigning diagnoses to DSM categories under explicit rules.

### Administrative codes.

Each diagnosis was first coded 0 (absent), 1 (present), 555 (not administered), or blank, with additional item-level codes (222, 444, 666, 777, 888, 999). We resolved every participant x session x diagnosis cell into one of four states: positive (1, criteria met), administered-negative (0, assessed but not met), not-administered (555), and no-record (blank). This yielded 18,005,108 cells (0.7% criteria met; 48.5% administered-negative; 37.7% not-administered; 13.2% no-record). Prevalence was computed only over administered cells (criteria-met plus administered-negative), so a not-administered cell was never counted as a negative.

### Episode status and clinical detail.

The tabulated data stores present and past diagnoses separately and distinguishes among current, past, partial-remission, and full-remission episodes. We retained each episode layer and defined caseness as either current (present only) or ever-met (present, past, or partial remission), with full-remission episodes kept as a separate layer and excluded from ever-met by default. Recurrence is not separately recoverable: the tabulated data distinguish present, past, and partial-remission episodes but contain no recurrent-episode variable. We also flagged whether each diagnosis is a subthreshold (“other specified” or “unspecified”) category, so that these can be included or excluded. The dataset works at the diagnosis level; item-level symptom data are not included but can be linked through the original variable names, which we preserve.

### Instrument version.

We tagged every cell with its KSADS-COMP version (1.0 for ses-00A through ses-02A, 2.0 from ses-03A on) and flagged diagnoses derived only in 2.0 that nonetheless appear under 1.0, so that version-sensitive analyses can be run or version-specific diagnoses excluded.

### Administration schedule.

For every combination of module, wave, and informant, we recorded whether the module was administered, not administered (recorded entirely as 555), or absent (not in the release at that wave). The resulting calendar denotes which not-administered cells are expected by design and which informant rules are available at each wave.

### Informant.

Parent and youth reports are marked independently in the resolved layer. The engine defines a case on the parent report, the youth report, either, or both. Where only one informant was assessed at a wave, the unavailable rules are flagged rather than treated as negative.

### Mapping to DSM categories and computing caseness.

We mapped the 230 diagnosis variables to 16 DSM categories (e.g., the six anxiety diagnoses to a single Anxiety category) and to broadband dimensions. From this, the caseness engine produces participant × wave × category status under four explicit choices: status (current vs. ever met criteria), informant (parent/youth/either/both), threshold (with or without subthreshold diagnoses), and construct membership (e.g., specific phobia in or out of Anxiety). A category is *positive* if any constituent diagnosis is positive, *administered-negative* if all modules were administered and none is positive and, otherwise, *not-administered*, preserving the administereddenominator rule at the category level.

## Data Records

The harmonized dataset is deposited in the NIMH Data Archive (NDA) as a derived data resource, available to investigators with an approved ABCD Data Use Certification. After logging in at https://nda.nih.gov, users can locate the dataset through the NDA Query Tool (“Get Data”) by searching the project title “Harmonized KSADS”, add it to their workspace and filter cart, and create a data package for download via the NDA Download Manager (https://nda.nih.gov/nda/nda-tools.html); the NDA interface is periodically updated, so users should consult current NDA documentation if these steps differ. We released the dataset in BIDS format. It includes the diagnosis-level data, with each cell labeled by state (positive, administered-negative, not-administered, or no-record), administration status, and version; category caseness tables for the default configuration; the administration calendar; and a table mapping each diagnosis to its DSM category, each with a JSON data dictionary.

## Technical Validation

### Cell-level validation.

Harmonization changes how each diagnosis is labeled, not whether it is present. Positive (criteria-met) cell counts match the raw ABCD release exactly (118,658 across all 16 categories). All 18,005,108 cells resolve to one of the four states with none unclassified; version tags align with the 1.0-to-2.0 switch at ses-03A; and no cell is scored positive or negative where its module was not administered (one off-schedule cell is retained and flagged; see Fig. 3B).

### Module-level validation.

Four modules screen positive at elevated rates even at full criteria: specific phobia (8.8%), OCD (7.7%), bipolar disorder (3.5%), and psychotic disorders (1.7%). We do not correct these; we flag them, so analysts inspect the constructs before use. The flag is externally corroborated: the instrument developers independently identified the same four modules and revised them between KSADS-COMP 1.0 and 2.0 to reduce false positives (a duration screen for OCD, the hypersexuality item moved to a bipolar supplement, and tighter hallucination and paranoia probes for psychosis). Given that ABCD’s baseline used 1.0, these baseline rates reflect the pre-revision instrument.

### Cross-wave validation.

Within-wave episode fields do not carry a participant’s own prior diagnoses forward. Among participants meeting criteria at one biennial wave, 42–70% have no record of that diagnosis at the next biennial wave (not current, past, or in remission) restricted to participants administered the module at both waves. The drop spans mood, anxiety, and externalizing modules; ADHD, which should rarely remit over two years, loses 42%, and OCD loses 70%. Within-wave “ever-met” therefore undercounts the cross-wave union of diagnoses, and lifetime status must be reconstructed across waves, as the harmonized longitudinal dataset does.

## Usage Notes

### Multiverse reporting.

We recommend reporting diagnostic cases across the range of defensible operationalizations, rather than at a single point. Given that the raw data support many analysis-ready variables rather than one, and each can give a different answer^11^, an analysis can be run under every reasonable specification and reported as a distribution, which shows how robust a result is and which choices it hinges on. To support this, we include a reporting checklist (Table 1). Diagnoses are provided individually (26) and grouped into 13 DSM categories (Table 2).

#### Clinically informed recommendation.

Some analyses need a single variable (covariate, matching factor, rate). The right decision largely depends on the research question; for example, decisions about status (current vs. ever met) and threshold (full criteria vs. including subthreshold/”other specified”) should follow from whether the analysis concerns point prevalence, lifetime risk, or subclinical presentation. That said, however, there are two choices that are not matters of preference: specifically, non-administration is always kept as missing, never recoded to 0, and the instrument version is always reported. In particular, “ever-met “criteria should be chosen deliberately and explicitly reported: past diagnoses far exceed current diagnoses at baseline (for major depression, 2.6% past vs. 0.2% current); thus, the ever-met option roughly doubles any-disorder caseness (47% vs. 25%), and these past episodes rest on retrospective report. For combining informants, parent and youth reports identify nearly independent sets of children (κ ≈ 0.02), so again, no single reporter can be treated as ground truth; rather than privileging one informant, we recommend reporting both single-informant rates and using the “either” rule^10^, noting that “either” raises sensitivity and should be labeled wherever a single rate is reported. Even with the default, sensitivity should be checked at the status and informant axes.

#### Specific phobia and the anxiety construct.

**Figure 5:**
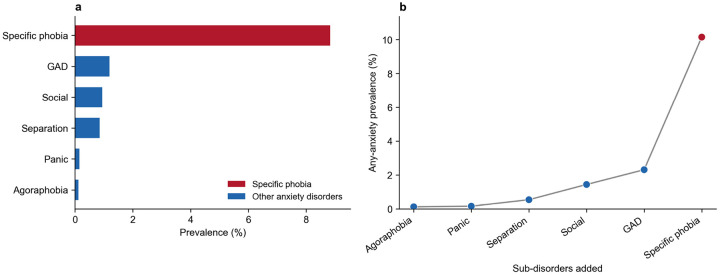
Anxiety group with and without specific phobia *Note*. (A) Baseline present-diagnosis prevalence (x-axis) for each anxiety sub-disorder (y-axis). (B) Cumulative “any anxiety” prevalence (y-axis) as sub-disorders are added one at a time along the x-axis, ending with specific phobia. The dashed line marks any-anxiety prevalence without specific phobia. All values are at baseline, computed over administered cells only.

One construct-membership choice warrants particular attention. The prevalence of specific phobia alone reaches 8.8%; including it raises any-anxiety prevalence from 2.3% to 10.2%, a 4.4-fold shift on identical data (Fig. 5). This is not specific to how the KSADS-COMP is administered in ABCD, but rather, reflects a long-recognized challenge for structured psychiatric interviews: separating a clinically significant phobia, which requires impairment or marked distress, from an “ordinary” childhood fear depends on clinical judgment that is not exercised consistently by trained lay interviewers. The effect is well documented. In the DISC, requiring attributable impairment markedly reduced the prevalence of diagnoses, with anxiety disorders among the most sensitive to whether impairment was applied^12^. For this reason, interviews such as the DISC, DAWBA, and CAPA built explicit impairment probes; the KSADS-COMP draws the fear-phobia distinction less stringently; its 2.0 revision added impairment criteria to several disorders, but notably, not to specific phobia. Consistent with this, specific phobia (administered at baseline, Year 2, and Year 4, then discontinued) declines smoothly across waves (8.8%, 6.0%, 4.1%), with no discrete drop at the version boundary. This gradual decline tracks development rather than the step changes a tightened criterion would produce. Whether specific phobia is included and/or grouped with other anxiety disorders should therefore be a deliberate, stated decision.

## Anxiety group with and without specific phobia

### Note

(A) Baseline present-diagnosis prevalence (x-axis) for each anxiety sub-disorder (y-axis). (B) Cumulative “any anxiety” prevalence (y-axis) as sub-disorders are added one at a time along the x-axis, ending with specific phobia. The dashed line marks any-anxiety prevalence without specific phobia. All values are at baseline, computed over administered cells only.

## Figures and Tables

**Table 1:** Reporting checklist for KSADS-COMP diagnostic definitions

Analytic decision	What to report	Recommended default	Include in sensitivity analysis?
Diagnostic status	Current vs. ever-met (present, past, remission)	Match to the research question	Yes
Informant	Parent, youth, either, or both	Either	Yes
Threshold	Full criteria vs. including subthreshold	Full criteria	Optional
Construct membership	Construct in or out of disorder group	State explicitly	Yes, especially for anxiety
Lifetime reconstruction	Whether ever met is computed within a wave or across waves	Union across waves for lifetime status	Yes, for longitudinal analyses
Administrative codes	How 555, 888, and missing values were handled	Not administered kept as missing; never recoded to 0	N/A
Instrument version	Which KSADS-COMP versions are included	Report version; flag the 1.0-to-2.0 transition	Yes, if analysis spans ses-03A
Administration schedule	Waves and modules used	Follow the administration calendar	N/A

*Note*. Each row is an analytic decision required to define a KSADS-COMP diagnosis. “What to report” is the choice an analyst should state; “Recommended default” is the configuration used when a single definition is needed; “Include in sensitivity analysis?” indicates whether varying the choice is advised (N/A where the choice is procedural rather than analytic). Status, informant, threshold, and construct membership correspond to the four axes varied in the specification curve (Fig. 1).

**Table 2:** DSM categories and their KSADS-COMP diagnoses

DSM category	Diagnoses (N)	Diagnoses
Anxiety	6	Generalized anxiety disorder; separation anxiety disorder; social anxiety disorder; panic disorder; agoraphobia; specific phobia
Eating	3	Anorexia nervosa; bulimia nervosa; binge-eating disorder
Psychosis	3	Schizophrenia; schizoaffective disorder; schizophreniform disorder
Tic	3	Tourette’s disorder; persistent tic disorder; provisional tic disorder
Depression	2	Major depressive disorder; persistent depressive disorder
Bipolar	2	Bipolar I disorder; bipolar II disorder
ADHD	1	Attention-deficit/hyperactivity disorder
ODD	1	Oppositional defiant disorder
Conduct	1	Conduct disorder
DMDD	1	Disruptive mood dysregulation disorder
OCD	1	Obsessive-compulsive disorder
PTSD	1	Post-traumatic stress disorder
Autism	1	Autism spectrum disorder

*Note*. Each row is a DSM category used in the harmonized dataset. “Diagnoses (N)” is the number of KSADS-COMP diagnosis variables mapped to that category, and “Diagnoses” lists them. Categories are ordered by the number of constituent diagnoses. A category is scored positive if any constituent diagnosis is positive (see Methods).

## Data Availability

The harmonized diagnostic dataset is deposited in the NIMH Data Archive (NDA), derived from Release 7.0 of the Adolescent Brain Cognitive Development (ABCD) Study. Both the harmonized dataset and the source release are available to investigators with an approved ABCD Data Use Certification; information on obtaining one is at https://www.nbdc-datahub.org/data-access-process.

## Code Availability.

Analytic code is publicly available on GitHub [https://github.com/eugiampetruzzi/abcd_ksads]. Analyses were run in Python 3.12 using pandas, numpy, and pyarrow.
